# The vagal paradox: A polyvagal solution

**DOI:** 10.1016/j.cpnec.2023.100200

**Published:** 2023-08-09

**Authors:** Stephen W. Porges

**Affiliations:** a Traumatic Stress Research Consortium, Kinsey Institute, Indiana University, Bloomington, IN, USA; b University of North Carolina at Chapel Hill, Chapel Hill, USA

**Keywords:** Polyvagal theory, Autonomic nervous system, Ventral vagal complex, Dorsal vagus, Engagement system, Threat reactions, Feelings of safety, Neuroception

## Abstract

Although there is a consistent literature documenting that vagal cardioinhibitory pathways support homeostatic functions, another less frequently cited literature implicates vagal cardioinhibitory pathways in compromises to survival in humans and other mammals. The latter is usually associated with threat reactions, chronic stress, and potentially lethal clinical conditions such as hypoxia. Solving this ‘vagal paradox’ in studies conducted in the neonatal intensive care unit served as the motivator for the Polyvagal Theory (PVT). The paradox is resolved when the different functions of vagal cardioinhibitory fibers originating in two anatomically distinguishable brainstem areas are recognized. One pathway originates in a dorsal area known as the dorsal motor nucleus of the vagus and the other in a ventral area of the brainstem known as nucleus ambiguus. Unlike mammals, in all ancestral vertebrates from which mammals evolved, cardioinhibitory vagal fibers primarily originate in the dorsal motor nucleus of the vagus. Thus, in mammals the vagus nerve is ‘poly’ vagal because it contains two distinct efferent pathways. Developmental and evolutionary biology identify a ventral migration of vagal cardioinhibitory fibers that culminate in an integrated circuit that has been labeled the ventral vagal complex. This complex consists of the interneuronal communication of the ventral vagus with the source nuclei involved in regulating the striated muscles of the head and face via special visceral efferent pathways. This integrated system enables the coordination of vagal regulation of the heart with sucking, swallowing, breathing, and vocalizing and forms the basis of a social engagement system that allows sociality to be a potent neuromodulator resulting in calm states that promote homeostatic function. These biobehavioral features, dependent on the maturation of the ventral vagal complex, can be compromised in preterm infants. Developmental biology informs us that in the immature mammal (e.g., fetus, preterm infant) the ventral vagus is not fully functional and myelinization is not complete; this neuroanatomical profile may potentiate the impact of vagal cardioinhibitory pathways originating in the dorsal motor nucleus of the vagus. This vulnerability is confirmed clinically in the life-threatening reactions of apnea and bradycardia in human preterm newborns, which are hypothetically mediated through chronotropic dorsal vagal pathways. Neuroanatomical research documents that the distribution of cardioinhibitory neurons representing these two distinct vagal source nuclei varies among mammals and changes during early development. By explaining the solution of the ‘vagal paradox’ in the preterm human, the paper highlights the functional cardioinhibitory functions of the two vagal source nuclei and provides the scientific foundation for the testing of hypotheses generated by PVT.

## Highlights

• Autonomic state functions as an intervening variable.
• A phylogenetically ordered response hierarchy that regulate autonomic state adaptation to safe, dangerous, and life-threatening environments.
• In response to a challenge, the ANS shifts states consistent with the Jacksonian principle of dissolution.
• Ventral migration of cardioinhibitory neurons leads to a ventral vagal circuit that supports an integrated social engagement system.
• Reflexive detection of risk (i.e., neuroception) triggers adaptive autonomic state to optimize survival.

## 1 Introduction

The initial presentation of the Polyvagal Theory (PVT) [1], proposed the use of evolution as an organizing principle to weave a descriptive narrative of the vertebrate phylogenetic journey towards sociality and co-regulation; a journey that documented a shift in the anatomical structure and function of the autonomic nervous system (ANS) and how these changes were involved in the mammalian biobehavioral features that enable co-regulation (e.g., mother-infant interactions) to support health and sociality. The intent was to use the scientific basis of the theory as a bridge to transform the broad mind-brain-body schisms in science into a more unifying perspective that incorporated an understanding of autonomic state as a neural platform that could support either sociality and feelings of safety or defensive strategies and feelings of threat.

As the theory gained traction in the scientific world, crossing several disciplines, and bridging basic science with clinical applications and personal experiences, the task of presenting a succinct statement of the theory became more difficult. Since the theory is dependent on several disparate disciplines, each with its specific literature, research questions, methodology, and theoretical orientation, the pragmatic task of communication has been fraught with complexity. This has created an intellectual challenge to accurately state the tenets of the theory and to convey its scientific foundation into constructs that are accessible and independent of academic background and profession.

The problem is further exacerbated as practitioners representing applied areas (e.g., medicine, education, business, and psychotherapy) have become interested in the theory and frequently convey elements of the theory to their constituencies, many of whom are not educated in the foundational sciences upon which PVT is dependent. The result has been a democratization of information on social media in which individuals may become influencers without having their academic credentials vetted and without the credibility of their claims being determined by the historical process of scholarly review. Unfortunately, given the complexity of the theory, the basics of the theory have not always been accurately transmitted, and misunderstandings can become misinformation within the digital world. This paper is an attempt to clarify the theory and rectify potential misunderstandings by documenting the scientific foundation upon which the theory is based.

## 2 Background: the vagus and the vagal paradox

The vagus is a cranial nerve that exits the brainstem and travels to several organs within the human body. It is the primary neural pathway of the parasympathetic nervous system. Functionally, the vagus is a bidirectional conduit between the brainstem and visceral organs. Although we generally focus on the motor functions of the vagus and how the motor pathways regulate the heart and the gut, the vagus is primarily a sensory nerve with approximately 80% of its fibers sending information from the viscera to the brain. The remaining 20% form motor pathways that enable brain circuits to dynamically and, at times, dramatically change our physiology, with some of these changes occurring within seconds. For example, vagal motor pathways can cause our hearts to beat slower and can stimulate our gut. Of these 20%, only a small percent is myelinated. Interestingly, the motor fibers dominate discussion of the role of the vagus in the regulation of the heart in biobehavioral and biomedical sciences (see Refs. [2,3]).

In its tonic state, the vagus functions like a brake on the heart's pacemaker (see Ref. [4]). When the brake is removed, the lower vagal tone enables the heart to beat faster. Functionally, the vagal pathways, regardless of brainstem nucleus of origin (i.e., dorsal or ventral) to the heart are inhibitory and slow heart rate. However, vagal cardioinhibitory actions are not solely chronotropic (i.e., influencing heart rate), but may have profound inotropic impact on contractility with consequential influences on heart rate through changes in blood pressure (i.e., baroceptors). Although the influence of inotropic vagal function is complex and not fully understood, recent studies document the important influences of cardioinhibitory inotropic vagal fibers originating from the dorsal motor nucleus of the vagus [[5], [6], [7], [8], [9]]. These studies describe the protective function of these pathways in the calm state as well as interactions with sympathetic inotropic influences. In addition, the literature documents experimental procedures during which the ionotropic impact of the vagus, the reduction of contractility, occurs independent of changes in heart rate.

In general, the synergistic effect of slowing heart rate and reducing contractility, is experienced as a calm state. Thus, vagal function is frequently assumed to be an “anti-stress” mechanism. However, there is another literature contradicting the positive attributes of the vagus and linking vagal mechanisms to life-threatening responses, such as bradycardia (and potentially hypotension through diminished contractility) that could lead to sudden neurogenic death (e.g., Ref. [10]). Basically, the same nerve, the vagus, proposed as a health supporting and anti-stress system, can stop the heart, reduce contractility, and lower blood pressure sufficiently to initiate syncope and, if prolonged, may lead to death (e.g., Refs. [11,12]). Convergent patterns of both positive and negative consequences of vagal excitation that have been observed in the gut have also been described as a ‘vagal paradox’ [13] Since the direct vagal input to the gut is primarily through the dorsal vagus, exploration of links between ventral vagal regulation of the heart and gut dysfunction may provide insights into the inotropic influences of the dorsal vagus on the heart.

## 3 The ANS regulation: antagonistic or hierarchical or both?

In virtually every text on anatomy or physiology, the ANS is described as a paired antagonistic system consisting of two opposing components. The texts generally describe a sympathetic nervous system that supports mobilized reactions to threat (i.e., fight and flight) and a parasympathetic nervous system that has the capacity to inhibit these debilitating and metabolically costly processes The net result of using this model may be described as a balance between these antagonistic systems (e.g., Ref. [14]).

In both clinical and research domains, terms like “autonomic balance” [15,16] have been used with an expectation that an optimal autonomic balance would be more parasympathetic (i.e., more vagal). This would be expressed as calmer and less reactive behavior. When vagal tone is depressed or withdrawn, we become tense and reactive and experience “stress.” This concise explanation of the role that the ANS and especially the vagus has in regulating our biobehavioral state is only partially correct. The story of how the vagus influences health and behavior is more complex. However, it is true that most of our visceral organs have neural connections from both the parasympathetic and the sympathetic nervous systems and that most parasympathetic neural fibers travel through the vagus.

The utility of this prevalent model breaks down in clinical investigations of high-risk human newborns in which vagal mechanisms are assumed to both support and compromise health. Insights from the high-risk newborn may further a reconceptualization contrasting the vagal mechanisms that support homeostatic functions with those that support threat physiology, especially during acute survival related challenges. There is a large literature documenting that the amplitude of respiratory sinus arrhythmia (RSA), a valid index of cardiac vagal tone [17], is related to positive clinical outcomes (e.g., Ref. [18]). In contrast, massive clinically life-threatening bradycardia also are assumed to be mediated by the vagus. Moreover, the preterm newborns with frequent bradycardia, who were at high risk for serious complications, reliably had low amplitude RSA (i.e., heart rate patterns with a relatively constant beat-to-beat rate) prior to a bradycardic event [[19], [20], [21]]. This contradiction in interpretation of vagal mechanisms form the basis of the **vagal paradox** posing the question: How could the vagus be both protective, when it was expressed as RSA, and life-threatening, when it was expressed as bradycardia and apnea?

Identifying the vagal mechanisms underlying the paradox evolved into the “Polyvagal Theory”. In developing the theory, the anatomy, development, evolutionary history, and function of the two vagal systems were identified: one vagal system mediating bradycardia and apnea and the other vagal system mediating RSA. One system was potentially lethal, while the other system was protective. The two vagal pathways originated in different areas of the brainstem. Through the study of comparative anatomy, it can be inferred that the two vagal circuits evolved sequentially (see Ref. [5]). This sequence was further observed during mammalian development (see Ref. [22]). Basically, hypotheses driven by PVT are related to the documentation that the mammalian ANS has a built-in **hierarchy of autonomic reactivity** based on phylogeny that is mirrored in embryological development. This fact became a core principle upon which PVT informed hypotheses could be tested. This emphasis on hierarchy is focused on ANS reactivity and does not preclude the optimal homeostatic states that involved a synergism and functional balance between parasympathetic and sympathetic influences. Thus, depending on the state of the ventral vagus, autonomic regulation may either function hierarchically or antagonistically.

## 4 ANS dependent distinctions between mammals and reptiles

Anatomical clues to PVT, especially those linked to social communication and connectedness, can be identified by investigating the three features that frequently are used to distinguish mammals from reptiles.

**Fig. 1:**
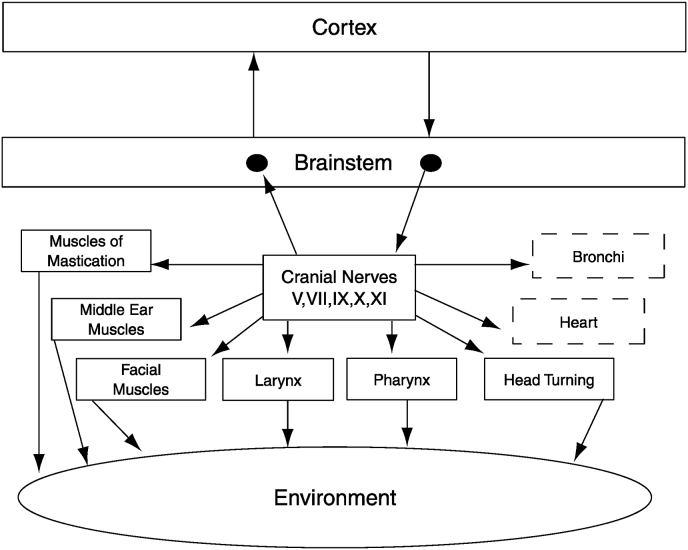
The social engagement system consists of a somatomotor component (solid blocks) and a visceromotor component (dashed blocks). The somatomotor component involves special visceral efferent pathways that regulate the striated muscles of the face and head, while the visceromotor component involves myelinated ventral vagal pathways that regulate the heart and bronchi.

First, mammals, as the name implies, have mammary glands, which provide milk to feed their young. This fact informs us that at birth mammalian offspring functionally can suckle [23]. From a polyvagal perspective, nursing is dependent on a functional **ventral vagal complex**, which enables the coordination of the ANS with the striated muscles to suck, swallow, breathe, and vocalize. The ventral vagal complex forms the neuroanatomical foundation of the **social engagement system** proposed in PVT and elaborated in the sections below (see Fig. 1). The operational definitions for the ventral vagal complex and the social engagement system are specific to PVT. These definitions do not preclude others from using similar terms that may include different anatomical structures supporting other behavioral functions.

The circuit also enables mammals to ‘broadcast’ their physiological state through vagal efferent fibers that control vocal intonation through pathways regulating laryngeal and pharyngeal muscles. The circuits regulated by the ventral vagal complex not only promote calm autonomic state via the ventral vagus, but also support several features embedded within maternal-infant interactions and sociality.

Second, mammals, unlike reptiles, have small middle ear bones that are detached from the jawbone. These small bones form an ossicle chain that functionally transmits the vibratory stimuli from the eardrum (i.e., tympanic membrane) to the inner ear. The middle ear muscles regulate the stiffness of the ossicle chain, which in turn controls the tension of the eardrum. When the eardrum is tightened the acoustic transfer function of middle ear structures dampens the acoustic energy of low frequencies and optimizes the transmission of frequencies associated with social communication (e.g., vocalizations). This evolutionary adaptation enabled mammals to detect airborne acoustic signals occurring at higher frequencies than those that detected by reptiles, whose acoustic processing was dependent on bone conduction. The ventral vagal complex also involves the nerves that regulate the middle ear muscles linking the extraction of prosodic vocalizations with the calming of autonomic state and social accessibility. In contrast, the low frequency roars of predators can trigger fight/flight reactions, while high-pitched screams trigger concern (see Refs. [24,25]).

This understanding of the adaptive function of middle ear muscles links listening to calming. It also provided the neurophysiological basis of an acoustic intervention known as the Safe and Sound Protocol™ (https://integratedlistening.com/products/ssp-safe-sound-protocol/). The Safe and Sound Protocol™ stimulates the ventral vagal complex to calm autonomic state, improve auditory processing, and stimulate spontaneous social behavior [[26], [27], [28], [29]].

Third, spontaneous heart rate-respiratory interactions, known as RSA in mammals, are dependent on myelinated vagal fibers originating in the ventral vagal nucleus in a brainstem region, known as nucleus ambiguus. This point distinguishes RSA from observations of respiratory-heart rate interactions in non-mammalian vertebrates and contributes to the maintenance of optimal physiological ventilation/perfusion [5]. This function may help explain the frequently noted power of RSA to predict various aspects of health.

## 5 Evolution: parallels between ontogeny and phylogeny

Evolution is used in PVT to identify the phylogenetic sequence of anatomical appearance and assumed adaptive function in vertebrates of brainstem structures involved in the regulation of autonomic state. The goal of this quest is to gain a better understanding of the structures that are expressed in the adaptive functions of the human ANS. To reach this goal there is an interest in the antecedents of these structures in the vertebrate species that evolved prior to mammals. Thus, PVT has a deep respect for continuities across vertebrate species. This respect for continuity is coupled with a focus on how repurposing the neural regulation of the ANS in antecedent vertebrates provided humans and other mammals with unique attributes enabling the regulation of the ANS to support sociality and down-regulate threat reactivity.

In mammals, ontogenetic changes in neural regulation of the ANS parallel phylogeny. Comparative anatomy leads the Polyvagal-informed scientist to investigate embryology and early development to confirm the maturational sequence in which neural structures regulate the ANS. The order of this sequence is important because the notion of a hierarchy, in which newer circuits inhibit older ones, is a core principle embedded in the history of neurology (e.g., Ref. [30]). The sequence ordering newer and older circuits is the same when mapped on a phylogenetic or ontogenetic timeline. The simplicity of the ontogenetic timeline is that this perspective is descriptive and does not require a dialog infused with hypothetical adaptive value or chronological time of emergence. PVT originated from the insights derived from using evolution as an organizing principle and metaphorically investigating the adaptive biobehavioral strategies of vertebrate species. However, PVT is only dependent on the identification of the sequence; a sequence that is also observed in the embryological development of humans and other living mammals [31].

PVT does not infer or identify the mechanisms through which evolution works. PVT treats evolution as providing a map of ancestral vertebrate relationships similar to a family tree. Theoretically, PVT is mammal-centric and is focused on the phylogenetic history of social mammals. PVT asks specific human-related questions, such as how does our evolutionary history inform our current understanding of human behavior and health? PVT focuses on the structural and functional changes in the mammalian ANS that relate to human experience. These questions differ from questions relating to modern reptiles. We share a common ancestor with modern reptiles, but we did not evolve from them. This point becomes of particular relevance as we explore the theory and especially how the theory may be misunderstood or misinterpreted.

## 6 Evolutionary transition from reptiles to mammals

To understand this evolutionary process, we need to have a better understanding of the timeline in which the transition from reptiles to mammals hypothetically occurred. Acknowledging the evolutionary timeline of mammals is critical in evaluating the relevance to PVT of neurophysiological research conducted with modern reptilian species (which evolved long after the earliest mammals). **Mammals did not evolve from modern reptiles**. Rather, the PVT emphasis on the evolutionary transition from reptiles to mammals refers to ancient and extinct reptiles that served as **common ancestors** for both mammals and modern reptiles. The common ancestor refers to the well accepted hypothesis that there was a long extinct reptilian species from which both modern reptiles and mammals evolved [32]. This point is critical, since it informs us that modern reptile species are NOT part of the phylogenetic history of mammals and are, therefore, irrelevant to PVT.

Modern reptiles are a product of an evolutionary journey that has shaped their anatomical structures, physiological functions, and behavioral strategies. This does not preclude consistencies between modern reptiles and mammals but acknowledges that there would have been major (presumably adaptive) changes during the estimated 220 million years since the emergence of both mammals and modern reptiles from the long extinct common ancestral reptilian species. To put this timeline into perspective, it is estimated that 200 million years is also the period between the earliest bony fish and mammals. Thus, inferences regarding modern reptile-mammal contrasts would need to be based on the hypothetical assumption that modern reptiles provide insights into features of this common, now long extinct, reptilian ancestor.

Millions of years before the existence of modern reptiles, the earliest mammals already had several features described by PVT. Since there is evidence that the earliest mammals could nurse [23], we can infer that, similar to modern mammals, they had a functional ventral vagus that was coordinated with the regulation of the structures of ingestion. If it were hypothetically possible to compare the earliest mammals with modern reptiles, these features would still be distinguishable even though 200 million years have elapsed.

## 7 Comparative neuroanatomy: limited inference

Comparative neuroanatomy helps identify the remarkable modifications in the regulation of the ANS in mammals that have resulted in an evolutionary trajectory providing the biobehavioral foundational building blocks of society – the ability to trust, feel safe, and co-regulate with conspecifics. These foundational processes recruit neural pathways that dampen threat reactions leading to emergent features of sociality that characterize most contemporary social mammals (see Refs. [33,34]). This does not preclude the importance of the evolutionary journey of modern reptiles, who occupy a niche different from that of social mammals in a complex dynamically changing and challenging world.

Comparative neuroanatomy does not document evolution but does infer evolutionary transitions from living species on which anatomical studies can be conducted. These extant species vary in their time of origin along the evolutionary timeline of vertebrates. In general, the fossil record has been used to date the time that specific species emerged. However, new molecular techniques, which were not available when the theory was proposed, frequently do not agree with the fossil record [35]. Although this contradiction is a challenge within comparative neuroanatomy, it is irrelevant to the basis of PVT, because the phylogenetic sequence relevant to PVT is mirrored in the embryology of contemporary mammals including humans.

Although a comparative perspective was instrumental in generating the working hypotheses that led to PVT, comparative neuroanatomy is not necessary or sufficiently conclusive to either support or disconfirm attributes of the theory. **Inferences regarding phylogeny can only be validated if the species being studied by comparative anatomists had not changed since their initial emergence.** Optimistically, if the brainstem structures providing the source nuclei for vagal pathways were studied in a reptilian species that did not change during the 200 million years since these lines diverged, then a better understanding of the transition from reptiles to mammals might be described. Of course, because evolution is not static nor linear, this assumption is too restrictive and impossible to achieve.

When PVT was first developed, the literature was scoured to determine whether it would be possible to study a reptilian species that evolved close to the time that mammals differentiated themselves from their reptilian ancestors. To do this, there is a need to estimate when a specific species evolved. Historically, the estimates have been based on the fossil record. However, with newer technologies, evolutionary biologists use a molecular time clock based on mutations in DNA to estimate age. Unfortunately, among reptilian species there is little convergence between the two methods. For example, turtle-like species, which had been assumed to represent an early reptile, using molecular methods appear to be more closely related to the more modern reptiles like crocodilians that evolved about 95 million years ago [35]. These inconsistencies disrupted the assumed phylogenetic timeline based on fossils that had been historically incorporated into evolutionary biology. Although we know that mammals and modern reptiles emerged from a common extinct reptilian ancestor, we can only cautiously talk about a timeline of evolution within this group of vertebrates. At this point, the timing is fluid of the exact phylogenetic sequence describing the lineage of reptilian species. This limits the use of comparative neuroanatomy in providing insights into the features of the common ancestor. Thus, beyond the phylogenetic sequence already described, it seems that comparative neuroanatomy and comparative neurophysiology have limited usefulness in refining PVT.

## 8 Ventral migration of cardioinhibitory neurons: the emergence of a social engagement system

The emergence of two vagal cardioinhibitory brainstem areas is a product of an evolutionary trend in ventral migration of cardioinhibitory neurons from the dorsal motor nucleus of the vagus to the ventral vagal nucleus (nucleus ambiguus). A trend towards ventral migration of vagal cardioinhibitory fibers is present in vertebrate groups that evolved before mammals [36]. **However, this research has little value to PVT and the study of mammals, since even with the earliest mammals, this migration was complete before modern reptiles evolved.** Not only does it appear that a ventral cardioinhibitory vagal nucleus is a defining feature of the earliest mammals, but, since it is assumed that the earliest mammals could nurse [23], the ventral cardioinhibitory vagus appears to have been integrated sufficiently with the regulation of the structures necessary for sucking. More simply put, the earliest mammals, but not reptiles, had already evolved the basic structures that are necessary for the nuanced social engagement system described in PVT, and which is based on the neural functions of the ventral vagal complex.

As the function of these two cardioinhibitory brainstem areas in mammals are investigated, an interesting narrative emerges about species differences in the distribution and function of the two cardioinhibitory areas. In certain reptiles, ventral migration of part of the original dorsal efferent cell column is observable in varying extents, from a simple ventral bulging of the cell column to a complete separation [36]. Although there is great uncertainty in the precise phylogenetic timeline of this migration in reptiles, we can assume that this migration was minimal within the long extinct reptilian species that predated the common ancestor. If correct this suggests that a major repurposing of cardioinhibitory wiring occurred in mammals relative to their ancient reptilian ancestors, allowing the integration of social engagement (via special visceral efferent pathways) with cardiovascular and ingestive demands. In fact, it is possible that this was a critical event in mammalian evolution.

The phylogenetic trend in the ventral migration of cardioinhibitory neurones can also be inferred from the study of mammalian development, especially through studies of embryology. This parallel had been acknowledged for decades (see Ref. [37]). An interesting interpretation of this developmental process has been reported in rats [38]. The latter study documented cardioinhibitory cells in three brainstem regions: dorsal motor nucleus of the vagus, ventral nucleus of the vagus, and an area between these two regions. The authors stated that the three locations appear to represent “no” migration, “complete” migration, and “abortive” migration which respective cardioinhibitory cell groups undergo during the embryonal stage. Nosaka and colleagues [38] speculated that the distribution of the cardioinhibitory neurons in mammals result from a variation in degree of **ventral migration** of these cells specifically determined for each species and potentially determining the autonomic substrate for the adaptive defensive behaviors they express. This speculation potentially explains observations of bradycardia as adaptively supporting immobilization in mammals that are prey species and are consistent with observations of bradycardia following electrical stimulation of the dorsal motor nucleus of the vagus in rabbits [1] and the spontaneous bradycardia which in rats can lead to death in response to life threat [10]. However, these chronotropic responses have not been observed in mammals that are predator species (e.g., dogs, cats), although there are reports of electrical stimulation of the dorsal motor nucleus of the vagus producing reduced contractility and lower blood pressure [[39], [40], [41], [42]]. This conclusion suggests that species (and even individual) differences in the function of each cardioinhibitory vagal nuclei might be dependent on success of ventral migration, which could be influenced during development by various processes (e.g., genetic variation, epigenetic modification, hypoxia, malnutrition, maltreatment, trauma, prematurity, illness, etc.).

As the cardioinhibitory neurons migrated ventrally, regulation of the structures that emerged from the ancient gill arches (facial and head structures in mammals) appear to have developed interneuronal connections with the ventral cardioinhibitory neurons. In mammals, the product of this brainstem neuroanatomical integration links the ventral vagal cardioinhibitory nucleus with nuclei that regulate sucking and social cueing via facial expression and vocalizations. Functionally, this neuronal circuit provided reliable pathways (e.g., vocalizations) to communicate autonomic state to conspecifics. **Developmentally, this is easily observed in humans because the social engagement circuit is active in full term newborns, creating an adaptive portal for co-regulation between mother and infant.**

Within PVT this network is called the “ventral vagal complex” (see Fig. 1). The ventral vagal complex is proposed as the neurophysiological substrate of an anatomically defined and functionally integrated **Social Engagement System**. **This system is neuroanatomically limited to the cranial nerve source nuclei from which specific special visceral efferent (i.e., branchiomotor) pathways emerge, although the afferent pathways traveling through the same cranial nerves constitute the afferent limb.** This system of interneuronal communication among these brainstem nuclei was forged by evolution and serves an important function in mammalian survival through its essential involvement of this system in ingestion and social communication.

**The Social Engagement System, based on a definable neuroanatomical substrate, supports the cooperative behaviors that differentiated the earliest mammals from ancestral reptiles.** The Social Engagement System in modern mammals continues to provide the substrate for co-regulation, attachment, and trust (i.e., processes through which social interactions regulate and optimize autonomic state to support homeostatic functions of health, growth, and restoration). This system, being based on the neuroanatomical structures involved in suck-swallow-breathe-vocalize pathways, has been described by others as a functional and defining feature of the earliest mammals [23].

Since the efferent pathways included in the social engagement system are exclusively special visceral efferent, it has been proposed that PVT has inappropriately excluded the hypoglossal nerve [43]. A deeper explanation of PVT notes that although the Social Engagement System is composed of special visceral efferent pathways, being classified as special visceral efferent is not the sole criterion for inclusion. Given that PVT has its roots in evolution, cranial nerves are viewed from an embryological and not solely from an anatomical perspective. In structuring the functional social engagement system and its anatomical substrate, the ventral vagal complex, the inclusion of specific special visceral efferent nerves was based on two criteria: 1) the nerve arises from pharyngeal arches during embryonic development, and 2) there is evidence of interneuronal communication between the nerve and the vagus. Applying these criteria resulted in clustering cranial nerves V, VII, IX, X, and XI, while excluding XII, the hypoglossal nerve. Consistent with these features, the sensory feedback into the motor centers regulating these specific special visceral pathways nerves may, via interneuronal connections, provide additional portals to regulate the ventral vagus and functionally may act as a vagal nerve stimulator.

Consistent with the emergence of a mammalian Social Engagement System, Theodosius Dobzhansky, a renown geneticist and evolutionary biologist [44] rephrased the concept of fitness by emphasizing in his description of mammals that “the fittest may also be the gentlest, because survival often requires mutual help and cooperation.” Dobzhansky's insightful statement converges on the emphasis of Polyvagal Theory on the phylogenetic transitions in neuroanatomy and neurophysiology as social mammals evolved from reptiles. Mutual help and cooperation are dependent on a nervous system that has the capacity to downregulate threat reactions to allow the proximity necessary for cooperative behaviors and co-regulation. In mammals this is neuroanatomically and neurophysiologically observed in the repurposed neural circuits originating in brainstem areas that regulate the ANS. The repurposed system enables feelings of safety to co-occur with sociality, allowing newborn mammals to engage with their mothers immediately following birth. This theme linking the ANS to sociality and feelings of safety has been elaborated in other publications (see Refs. [33,34].

## 9 Monitoring development of the ventral vagus via RSA

In humans, the embryology literature suggests a maturational progression similar to the phylogenetic trend inferred from comparative neuroanatomy (see Ref. [22]). Since the preponderance of myelinated cardioinhibitory vagal fibers originate in the ventral vagal nucleus, and not the dorsal motor nucleus of the vagus, there is the opportunity to map the ventral migration through autopsy data detailing the distribution of myelinated and unmyelinated vagal fibers. Autopsy data [45,46] confirm a developmental increase in the number and ratio of myelinated vagal fibers. Moreover, there seems to be a decrease in the survival rate of infants who have an apparent deficiency in myelinated vagal cardioinhibitory fibers. This deficiency has been reported in infants who have died from sudden infant death syndrome, a disorder assumed to be associated with neurogenic bradycardia [47]. Thus, although there may be phylogenetic antecedents of a convergent evolution (see Ref. [48]) of myelinated cardioinhibitory fibers originating from the dorsal motor nucleus of the vagus [49], the consensus view is that within mammals the predominant cardioinhibitory influence from the ventral vagal nucleus is conveyed through myelinated fibers. The functional output of the ventral vagal nucleus follows a maturational trend. When there is a deficiency in the number of myelinated cardioinhibitory fibers originating in the ventral vagal nucleus there may be a lower threshold (see dissolution below) to neurogenic bradycardia (potentially augmented by ionotropic influences) through the unmyelinated cardioinhibitory fibers originating in the dorsal motor nucleus of the vagus (e.g., Ref. [47]). The latter point is consistent with PVT.

Since the fibers emerging from the ventral vagal nucleus have a respiratory rhythm [5,50,51], it is possible to track the functional impact of these pathways through early development by studying RSA in laboratory mammals (e.g., rats, rabbits) and preterm infants. PVT limits the definition of RSA to the respiratory-heart rate pattern observed in mammals that is a function of myelinated vagal fibers originating in the ventral vagal nucleus (nucleus ambiguus). Historically, the term RSA has been used solely to describe the pattern observed in mammalian species. Describing respiratory-heart rate patterns in other vertebrates does not mean that the neural mechanisms are identical to those observed in mammals. In fact, in vertebrate species other than mammals, except for the report of a myelinated cardioinhibitory pathway emerging from the dorsal motor nucleus of the vagus in the lungfish [49], all reports document that heart rate-respiratory interactions were mediated via unmyelinated vagal cardioinhibitory pathways originating in the dorsal motor nucleus of the vagus. The identification of the myelinated fibers in the lungfish have been misused to infer a ‘fatal flaw’ in PVT. However, the identification of myelinated vagal fibers in lungfish is unrelated to PVT and reflects a misunderstanding of PVT. The lungfish appears to be a phylogenetic outlier, having vertebrate ancestry that did not have myelinated cardioinhibitory dorsal vagal fibers nor has this feature been reliably transmitted to the groups of vertebrates that subsequently evolved (i.e., amphibia, reptiles, mammals).

Larson and Porges [52] described the development of RSA in rat pups. Rats have a short gestation and are born extremely premature relative to humans. Their study documented a maturational trajectory of increased RSA. At birth and during the first few days of life, postpartum RSA was negligible, although by day 20 it had reached the level of adult rats. A study with fetal sheep [53], documented that as the fetus matured, even in the absence of systematic fetal breathing movements, the pattern of RSA became more sinusoidal, potentially reflecting the maturational increase in the myelination of the ventral vagal cardioinhibitory pathways.

Consistent with the animal research, our human research documented significantly lower amplitude RSA in preterm relative to full-term newborns [18,54]. In addition, maturational RSA trajectories in high-risk preterm newborns were observed [55] and RSA was enhanced in preterm newborns through social engagement opportunities with caregivers [56]. These human infant studies, consistent with PVT, document that the maturation of the ventral vagal cardioinhibitory circuit can be monitored through the measurement of RSA and can be optimized through social engagement opportunities that may function as neural exercises involving the ventral vagal complex.

## 10 Dissolution

The identification of this phylogenetic sequence provides the generation of testable PVT-informed hypotheses linked to the Jacksonian principle of dissolution [30]. Embedded in dissolution are the following points: 1) there is a phylogenetic as well as an ontogenetic hierarchy, in which newer circuits inhibit older circuits; and 2) during responses to brain illness or damage to the function of brain structures, changes occur in a predictable sequence that has been described as dissolution or evolution in reverse.

PVT broadens the Jacksonian principle to examine its application not only to changes in higher brain structures, but also in foundational survival-based brainstem structures that regulate the ANS. In addition, PVT recognizes the parallel trends expressed in both the ontogeny and phylogeny of the mammalian ANS. Thus, dissolution is expressed as development in reverse, basically hypothesizing that the more ancient (and earliest maturing) autonomic regulatory circuits (i.e., sympathetic nervous system and dorsal vagal) would sequentially be disinhibited during prematurity to optimize survival. With either an ontogenetic or phylogenetic bias defining dissolution, we arrive at the same plausible and testable hypothesis - under challenge there is a progression that could be characterized as either evolution or development in reverse. Thus, the phylogenetic sequence or its equivalent maturation sequence would unfold in reverse in response to life challenges, whether pathogen, physical injury, or anticipation of life threat (e.g., predator). **By focusing on dissolution through a developmental lens, grounded in research from embryology and early postpartum development, we avoid the phylogenetic, and often untestable, distractions that may lead to misunderstandings of PVT.**

## 11 A test of PVT: dissolution in the neonatal intensive care unit

In earlier research Reed et al. [57] reported a relationship between RSA and a vulnerability to life-threatening bradycardia during delivery. Below is a portion of the abstract, which succinctly describes the pattern of dissolution predicted by PVT and supported by the developmental and phylogenetic literature.

*Transitory****heart****rate****accelerations****and****reduced beat-to-beat variability****reliably preceded heart rate decelerations. The data are interpreted within the context of the Polyvagal Theory, which provides a plausible explanation of the neurophysiological mechanisms that mediate fetal heart rate decelerations. Specifically, it is proposed that both the transitory heart rate accelerations and the depression of the respiratory rhythm in the beat-to-beat heart rate pattern reflect a withdrawal of the vagal tone determined by myelinated vagal pathways originating in the nucleus ambiguus. Functionally, withdrawal of vagal tone originating in the nucleus ambiguus results in the cardiac pacemaker becoming vulnerable to sympathetic influences and to the more-primitive unmyelinated vagal pathways originating in the dorsal motor nucleus of the vagus, which may contribute to clinically relevant bradycardia. Developmental Psychobiology 35:108–118, 1999*

The above explanation of the adaptive functions of the two vagal pathways provides documentation of an empirical solution to the vagal paradox. Whether this sequence is expressed in mature mammals is an empirical question. It is possible that the chronotropic influences via dorsal vagus are minimized with maturation, although ionotropic influences may persist and may contribute to neurogenic bradycardia through the ventral vagus. With appropriate research questions and methodologies, this question can be answered.

This does not preclude the special heuristic case of the preterm human newborn, who may be at a point in development during which ‘ventral’ migration and myelinization of chronotropic vagal neurons are active processes. Thus, the clinical bradycardia observed in the neonatal intensive care unit could be mediated through dorsal vagal pathways. Documentation for this possibility comes from the observation that chronotropic influence through ventral vagal pathways distinctly has a respiratory rhythm [5], while chronotropic influences through the dorsal vagal pathways do not. In the preterm newborn study [57], the background heart rate upon which the bradycardia is observed is devoid of a respiratory rhythm. In fact, the prevalence of bradycardic episodes was directly related to periods during which RSA was suppressed.

## 12 Dorsal vagus through the lens of the PVT: an update

Perhaps, the focus on vagal chronotropic influences via dorsal vagal pathways, which reliably may be observed in the immature newborn but not the mature adult, has distracted from a potential contribution of the inotropic mechanisms that involve neurons in the dorsal motor nucleus of the vagus. It is possible that during periods of sufficient ventral vagal control to support homeostatic functions (i.e., health, growth, and restoration), the inotropic influence on the ventricles, via the dorsal vagus, would be protective. However, in the absence a strong ventral vagal influence, consistent with dissolution, there may be a vulnerability for inotropic influences via the dorsal vagus to be disruptive to blood pressure regulation, which under certain cases might recruit ventral vagal pathways in producing life threatening bradycardia.

The possibility that myelinated ventral vagal pathways might contribute to bradycardia during conditions of compromise has been suggested as being consistent with PVT [58]. Basically, it is possible that dorsal vagal mechanisms may threaten survival by disrupting blood-gas status sufficiently to depress respiratory and blunt RSA, while triggering a compensatory bradycardia through ventral vagal pathways. Future research will need to determine whether this is a viable hypothesis.

It is important to note that the dorsal vagus has beneficial functions in humans. During most normal conditions, the dorsal vagus maintains tone to the gut and promotes digestive processes. However, if up regulated, the dorsal vagus contributes to pathophysiological conditions including the formation of ulcers via excess gastric secretion and colitis. This leads to an important question that might explain the inotropic reactions within a PVT perspective. PVT emphasizes the hierarchical sequence based on evolution and development to develop hypotheses of autonomic regulation in response to challenges. In building the theory, the insights came from the readily observable clinical tachycardia followed by bradycardia in preterm newborns who had depressed RSA. Depressed RSA in preterm newborns is due to the prematurity of their ventral vagal pathways and not a function of illness or stressful contexts, although these factors could further compromise post-partum development. Consistent with PVT, the depressed ventral vagus provides an opportunity for sequential shifts in autonomic regulation involving both sympathetic and dorsal vagal pathways.

PVT proposes that when the ventral vagus is optimally managing a resilient autonomic nervous system both the sympathetic and dorsal vagus are synergistically coordinated to support homeostatic functions including health, growth, and restoration. However, when ventral vagal influences are diminished, as index by depressed RSA and overall heart rate variability, then the sympathetic and dorsal vagal pathways are poised to be sequentially recruited for defense. Autonomically, this would be observed initially as increased heart rate and cardiac contractility, while suppressing the inhibitory calming and homeostatic actions of the dorsal vagus on the heart and gut. Since the sympathetic defense strategy is metabolically costly, the dorsal vagal influence on the heart and gut may be triggered as a metabolically conservative, defensive surge expressed as reduced contractility of the heart, a lowering of blood pressure, and a clearing of the bowel. In general, the literature on RSA and heart rate variability suggests that a depressed RSA is a covariate for several health conditions including types of gut dysfunction, which have been assumed to be mediated by dorsal vagal influences.

## 13 Repurposing the dorsal vagus: species specificity

The specificity of mammalian species repurposing the function of the dorsal vagus on the heart (minimizing chronotropic, while maintaining or potentially enhancing ionotropic functions) does not appear to modify the roles of the dorsal vagal pathways regulating the subdiaphragmatic organs (e.g., gut), which may contribute to the high prevalence of clinical reports of irritable bowel syndrome in survivors of trauma even if they had not immobilized. Although bradycardia may not be a reliable phenomenon for all who experience life threat other autonomic changes mediated via the dorsal vagus, such as reduction in contractility and gut reactions may be more reliable indicators of a threat reaction. Thus, while species differences were accounting for some of the confusion in the literature relating the chronotropic cardioinhibitory role of the two vagal nuclei [42], the important ionotropic role of dorsal vagal pathways may have been neglected.

A full understanding of cardioinhibitory function via vagal pathways in humans is still speculative. However, it may reflect a range of individual differences that could be broadened by potential disturbances to normal development by perinatal challenges, such as prematurity, hypoxia, maltreatment, and malnutrition. Although it has been assumed that in humans the vagal chronotropic fibers traveling from the dorsal motor nucleus of the vagus to the heart are functionally dormant [59], this assumption may not be accurate. Potentially, this system may be sensitive to or reserved for action in response to life-challenging signals such as hypoxia or alternatively the dorsal vagal inotropic pathways may trigger, via changes in blood pressure, ventral vagal chronotropic reactions. Thus, hypothetically, this neural circuit may map into a range of individual differences that would parallel the clinical observations of individual variations in the propensity to shut down or even faint in response to threat. Research suggests that in a normal ‘homeostatic’ state, dorsal vagal pathways have a protective ionotropic role on the myocardia (See Refs. [[5], [6], [7], [8], [9]]). However, it is possible that during threat there may be an acute increase in inotropic influence that would be sufficient to trigger hypotension and syncope.

## 14 The vagal brake: a measure of ventral vagal efficiency (VE)

### 14.1 Ventral vagal brake

In humans, the ventral vagal efferent pathways to the heart function as a brake. The intrinsic rate of the heart in the healthy human, even without sympathetic excitation, is significantly faster than the resting heart rate. Thus, under most conditions, the vagus, primarily via myelinated vagal fibers originating in the nucleus ambiguus, actively inhibits heart rate. However, when there is a need to engage actively with select elements in the environment, cortical neurons inhibit homeostatic needs, and cardiac output is rapidly increased to match metabolic demands. Under these situations there is a transitory withdrawal of the vagal tone to the heart to increase heart rate, which defines the removal of the vagal brake [4].

The vagal brake reflects the inhibitory influence of the myelinated ventral vagal pathways on the heart, which slows the intrinsic rate of the heart's pacemaker. The intrinsic heart rate of healthy adults is about 90 beats per minute. However, in humans baseline heart rate is noticeably slower due to the influence of the ventral vagus, which functions as a “brake.” When the ventral vagus decreases its influence on the heart, the “brake” is released, and heart rate spontaneously increases. This is not solely due to an increase in sympathetic excitation, rather, the release of the vagal brake allows the intrinsic rate of the pacemaker to be expressed. The vagal brake represents the actions of engaging and disengaging the ventral vagal influence on the heart's pacemaker. In addition, the release of the vagal brake on the heart also enables tonic underlying sympathetic excitation to exert more influence on the autonomic nervous system. PVT [1,4,60,61], specifically assumes that the vagal brake is mediated primarily through the myelinated ventral vagus and can be quantified by the amplitude of RSA. The theory acknowledges other neural (e.g., dorsal vagal pathways) and neurochemical influences that can influence heart rate (e.g., clinical bradycardia), but these mechanisms are not involved in mediating the chronotropic influences of the ventral vagal brake as defined within PVT.

The vagal brake is conceptualized as an adaptive neural physiological mechanism that fosters engagement and disengagement with the environment. When demands require a calm behavioral state, the reengagement of the vagal brake slows heart rate and provides the physiological support for self-soothing behaviors. There is a large literature documenting the baseline or non-challenge level RSA and other metrics of heart rate variability are related to mental health outcomes with higher values usually being associated with more positive outcomes and greater resilience (See Ref. [62]). When the vagal brake efficiently supports the changing metabolic demands, the neural modulation of RSA is paralleled by a monotonic change in heart rate.

### 14.2 Ventral vagal efficiency (VE)

The efficiency of the vagal brake might be evaluated along several dimensions, including changes in the amplitude of RSA or an index of heart rate change relative to RSA change in response to a defined challenge. The definition of a challenge is arbitrary and often defined within specific experimental paradigms (e.g., mental effort, attention, sleep state, exercise, social interaction, posture shift). Especially during alert or vigilant states, responses to challenges must be rapid and continuous. For example, environmental demands often dynamically change under real life conditions.

To evaluate the dynamic function of the vagal brake, it is necessary to generate measures of RSA and heart rate for short sequential epochs. Most methods for quantifying RSA, such as spectral analysis have assumed inappropriately that the amplitude of RSA was a stationary characteristic of the heart rate time series. In general, these methods require periods of several minutes to calculate an average amplitude of RSA. Measurement over longer periods of time assumes that the variations over shorter periods of time are statistically treated as measurement error. However, to evaluate the dynamic function of the vagal brake, the estimates for heart rate and RSA need to be calculated in periods or epochs of short durations.

Epoch-by-epoch shifts in RSA can be evaluated as a measurable manifestation of dynamic changes in the vagal control of the heart and not assumed to be measurement error distributed around a central tendency. Therefore, it would be necessary to quantify RSA over relatively short periods of only a few seconds. Unlike other methods, the Porges-Bohrer method [17,63,64] provides an opportunity to study the dynamically changing amplitude of RSA independently of a potential nonstationary baseline representing dynamic changes in heart rate.

The efficiency of the vagal brake might be evaluated along several dimensions, including an index of heart rate change relative to RSA change in response to a defined challenge. This metric, now labeled vagal efficiency (VE), describes the dynamic relationship between RSA and heart rate. In several papers we have measured VE during a posture challenge, since it reflexively shifts vagal influences on the heart, is independent of cognitive or social demands, and is easily standardized. Moreover, it provides an opportunity to incorporate a manipulation that involves dynamic challenges involving barosensory feedback. VE is calculated as the slope of the linear regression between synchronous pairs of short duration epochs (e.g., 15 s) values of RSA and heart period monitored during the posture conditions (e.g., supine, sit, stand). VE measures the dynamic effect of on heart rate as the instantaneous coupling between RSA and heart rate. VE is quantitatively assessed by measuring the slope of the linear regression between short epoch estimates of heart rate and RSA. The slope is easily interpreted as the magnitude of heart period (reciprocal of heart rate) change in ms per unit of RSA amplitude. The steeper the slope, the greater the impact or efficiency of the vagal brake on heart rate. In those who have high VE scores, RSA changes produce similar impact on in heart rate independent of actual RSA. Low correlations between RSA and VE further support this observed statistical independence.

Previous research demonstrates that VE degrades in response to alcohol [65], preceding death following surgery in prairie voles [66], differentiates sleep states in healthy newborns [67] with greater VE during active sleep when cardiac-somatic coupling would be required, and exhibits a maturation shift post-partum in high-risk newborns [56]. Studies evaluating VE during posture challenges documented that it was low in adolescents with joint hypermobility syndrome [68], low in patients with functional abdominal pain [69] and was not influenced by partial cholinergic blockade (unpublished). The latter finding suggests that VE is reflecting the status of brainstem integrative circuits and may provide information that is not observed in RSA as a measure of cardioinhibitory vagal outflow. Moreover, the data suggest that the metric is useful even when the range of heart rate and RSA are not extended through metabolic or barosensory challenges. Psychometrically, this would be consistent with the report that distributions of regression line slopes are relatively immune to the influence of range [70].

In preliminary research, we explored the relationship between VE and maltreatment history. We investigated whether the atypical patterns of autonomic reactivity and recovery to stressors frequently observed in survivors of trauma is influenced by an inefficient vagal brake [71]. The study documented that maltreatment histories were associated with lower VE, which in turn mediated more anxiety and depression symptoms. VE, by reflecting a disruption in feedback between the heart and brainstem that may also lead to body numbness, could index autonomic regulation to stressors and psychiatric symptomatology. Blunted VE may be a mechanism through which maltreatment induces mental health risk and interventions aimed at promoting efficient vagal regulation may be promising for improving resilience and wellbeing in trauma survivors. In summary, VE may be a powerful, low cost, easily quantifiable, and scalable measure that would potentially provide rapid throughput screening that would identify a ventral vagal parameter of atypical autonomic regulation. The VE metric might contribute to a refined diagnoses of dysautonomia and several functional disorders.

## 15 Body perception questionnaire: self-reported autonomic reactivity

Polyvagal Theory emphasizes that autonomic state is an intervening variable mediating individual differences in responding and recovering from challenges. In general research testing hypotheses generated by the theory have been dependent on monitoring autonomic variables, especially indices of ventral vagal influence such as RSA and VE. However, a dependence on physiological monitoring would limit the ability to test hypotheses outside of well-equipped laboratories. In response to a need to assess autonomic state regulation in survey research, we developed a questionnaire, the Body Perception Questionnaire Short Form (BPQ-SF) [[72], [73], [74], [75]]. The BPQ-SF [72] provides a measure of self-reported experiences of reactivity in organs and tissues that are regulated by the ANS. The BPQ-SF has been found to have good psychometric properties, convergent validity with similar measures, and consistent factor structure across samples [73]. In a laboratory validation study [75] higher scores of autonomic reactivity on the BPQ-SF were associated with destabilized autonomic reactivity patterns (i.e., lower RSA, higher heart rate, poorer recovery to challenge).

In another study using the BPQ-SF, the frequently reported association between sexual function and adversity history was mediated by an ANS biased toward maintaining a physiological state that supports defensive strategies [76]. Consistent with the above relationship, we documented that the BPQ-SF measure of autonomic reactivity mediated the relationship between adversity history and mental health symptoms during the early phase of the Covid-19 pandemic (March 29 to May 13, 2020) in individuals who had not been infected [77]. These studies confirm that a self-report measure of autonomic regulation can be used in survey research as a reliable intervening variable in mediating the impact of adversity on outcomes (e.g., mental health, sexual function) and provide a tool to inexpensively test Polyvagal informed hypotheses without the burden of physiological monitoring.

## 16 Neuroception

Polyvagal Theory proposes that the neural evaluation of risk and safety reflexively triggers shifts in autonomic state without requiring conscious awareness. Thus, the term “neuroception” was introduced to emphasize a neural process, distinct from perception, capable of detecting and distinguishing environmental and visceral features that are safe, dangerous, or life-threatening [78,79]. In human and other social mammals, neuroception is conceptualized as ‘reflexive’ reactions that prepare the organism for defense or inhibits defense to promote homeostatic functions including health, growth, restoration, and sociality. A form of neuroception can be found in virtually all living organisms, regardless of the development of the nervous system. In fact, it could be argued that single-celled organisms and even plants have a primordial form of neuroception that responds to threat. As mammals, we are familiar with reactions to pain, a type of neuroception. We react to pain prior to our ability to identify the source of the stimulus or even of an awareness of the injury. Similarly, the detection of threat appears to be common across all vertebrate species. However, mammals have an expanded capacity for neuroception in which they not only react instantaneously to threat, but also respond instantaneously to cues of safety. It is this latter feature that enables mammals to downregulate defensive strategies to promote sociality by enabling psychological and physical proximity without an anticipation of potential injury. It is this calming mechanism that adaptively signals the central regulation of autonomic function to dampen the metabolically costly fight/flight reactions dependent on sympathetic activation and to protect the oxygen-dependent central nervous system, especially the cortex, from the metabolically conservative defensive reactions of the dorsal vagal complex (e.g., fainting, death feigning).

PVT proposes that neuroception functionally involves both top-down and bottom-up mechanisms. The process of neuroception is assumed to be initiated via top-down pathways involving cortical areas located in or near temporal cortex, components of the central nervous system that reflexively interpret cues of threat and safety. These areas of the cortex are sensitive to the intentionality of biological movements including voices, faces, gestures, and hand movements. Embedded in the construct of neuroception is the capacity of the nervous system to react to the intention of these movements. Neuroception functionally decodes and interprets the assumed goal of movements and sounds of inanimate and living objects. Thus, the neuroception of familiar individuals and individuals with appropriately prosodic voices and warm, expressive faces frequently translates into a positive social interaction, promoting a sense of safety (for example, safety cues in mothers’ voices reduce infant heart rate and behavioral distress [80]).

Autonomic state responds to the top-down detect of risk or safety. The autonomic reactions send sensory information regarding bodily feelings to the brain where they are interpreted and consciously felt. The bottom-up limb of the neuroception is functionally equivalent to interoception. Thus, although we are often unaware of the specific features of the stimuli that trigger neuroception, we are generally aware of our body's reactions (i.e., visceral feelings) embodied in autonomic signatures that support adaptive behaviors (i.e., social engagement, fight/flight, shutdown).

## 17 Polyvagal theory: principles

As listed in the table below, PVT can be summarized in five primary principles. Although the principles are succinctly stated, they reflect an extraction of the complex interdisciplinary material presented in the preceding sections as well as the wealth of information that has accumulated since the theory's initial presentation in 1994. During that period, PVT has been cited in more than 15,000 peer reviewed journals and unexpectantly, thousands of therapists currently self-identify as being Polyvagal informed.

The principles form an interdependent hierarchical model in which each principle needs to be acknowledged sequentially. Feedback from both the research and clinical communities have helped provide clarity in articulating these principles as the theory evolved during the three decades since its initial presentation. In its original form the theory had a speculative hypothetical focus derived through extracting principles from the literature. Literally, the initial presentation was structured as a challenge to colleagues to expand, refine, or refute features of the presentation with an optimistic and collaborative goal of gaining a better understanding of how autonomic state was related to human experience.

When I introduced the theory in 1994, as a laboratory scientist, I had limited experience in the realm of mental health and especially in the now burgeoning field of trauma. By the late 1990s I was presenting the theory at meetings for mental healthcare providers often with a focus on trauma. To my surprise at these meetings, I was informed by survivors of severe adversity that PVT provided them with a narrative to explain their personal experiences. I was also being informed by several therapists that the first thing they did with their trauma patients was to explain PVT. Through these experiences in the clinical world, I witnessed an intuitive validity and utility of PVT as a scientific biobehavioral narrative that was consistent with the experiences of trauma survivors. Moreover, both therapists and survivors personally informed me about the therapeutic power of understanding that their reactions were neurobiological ‘reflexive’ scripts of survival outside the realm of intentional behavior. This shifted their understanding of their own experiences from shame and blame (e.g., why didn't they run or fight) to a deep respect for their body's foundational survival mechanisms that were dependent on brainstem circuits regulating the ANS.

### 17.1 Overview: PVT as an algorithm

Given the background of having approximately 30 years of feedback from both the research and the clinical communities, the challenge is whether the principles of PVT can be succinctly refined to be sufficiently accessible in a manner that is respectful of both its scientific basis and the experiences reported in the clinic. To do this, we need to delve into the roots of the theory and the fundamental questions that it addresses.

Polyvagal Theory proposes that specific features of autonomic function in mammals are recruited to optimize survival. This is far from an innovative proposition. However, PVT proposes that this hypothetical ‘optimal survival’ is the product of a functional neural algorithm through which the nervous system makes survival related decisions based on a variety of factors. Like any decision-making entity, there is an acknowledgement of three sources of information (i.e., input, output, internal ‘processing’). In this model input is the challenge, output is the response, and internal processing is conducted by the nervous system.

Historically, in its dedicated search for ‘laws of nature,’ science primarily focuses on only two of these sources (cause and effect or stimulus-response), while treating the internal resources of the entity (i.e., organism) as random error. This is the case in the application of Randomized Control Trials (RCT), the gold standard in medical research. However, if we include features of autonomic resilience, such as its individual or situational capacity to efficiently recover from disruptive challenges to support homeostatic functions, then the model expands from testing cause and effect hypotheses to questions of how neural regulation of ANS mediates reactivity and recovery to challenges. In today's data-oriented world, this neural algorithm would be conceptualized as a mediational model that would generate ANS signatures (profiles) to optimize processes across a wide range of adaptive adjustments from those supporting the body's homeostatic processes (health, growth, and restoration) to the metabolically costly survival processes demanding efficient fight and flight actions. This mediational algorithm contrasts with the cause-and-effect inference extracted from a RCT model or epidemiology's reliance on linear models to infer cause-and-effect relationships.

PVT explores the implication of this mediational algorithm. This would enable science to provide an index of different autonomic states that would provide autonomic signatures useful for a variety of disciplines (i.e., monitoring accessibility to learn, to socialize, to support homeostatic processes of health, growth, and restoration). Perhaps, the most informative aspect of such an algorithm would be to identify the autonomic pathways that would support the ability to down regulate threat to enable mobilization and immobilization to occur with trusted others and not trigger defense. For example, the algorithm could be applied to confirm whether specific autonomic pathways are recruited to support apparently contradictory demands that require mobilization such as play in contrast to fight-flight behaviors or immobilization such as intimacy in contrast to death feigning. **It is this process of functionally liberating mobilization and immobilization from defensive threat driven strategies that PVT hypothesizes to have supported the emergence of social behavior and cooperation in species of social mammals** [33,34].

### 17.2 Principle 1. autonomic state functions as an intervening variable

Within this concept of an algorithm how do the theory's principles fit? Principle 1 focuses on the resource and flexibility of the system – the capacity to respond, process, and recover. Functionally this principle emphasizes that autonomic state serves as a ‘neural’ platform that limits and fosters broad domains of behaviors and psychological experiences during contexts of safety, danger, and life threat.

PVT expands and diverges from the neuroscience disciplines studying parallels between autonomic state and mental, physical, social, affective, and health processes. PVT encourages researchers to go beyond conducting studies to confirm these correlations. PVT emphasizes the limitations of correlational research as an imprecise research strategy that obfuscates the critical mediating mechanisms of the correlated phenomena. PVT views correlational research as focusing on the phenomena that covary and not the underlying neural pathways that would explain how autonomic mechanisms would be integrated within the actual processes being studied. PVT emphasizes an important perspective missed by correlational research - how the ANS is part of an integrated response and not a covariate. This might reframe the current use of co-morbidities within mental and physical health diagnostic systems from correlative relationships to being an attribute of a disrupted system that could be expressed in several outputs including features of both mental and physical health.

Historically, PVT is consistent with the work of Ernst Gellhorn [14], who emphasized the integration of autonomic, cortical, and somatic systems. In contrast, correlational based sciences such as epidemiology provide probabilities that are frequently interpreted as insights into causal mechanisms and often, due to faulty inference, lead to inappropriate treatments with poor outcomes.

Once the research target shifts from correlation to parameters mediating the integration, then the diverse components of the nervous system, including somatic, cortical, autonomic, endocrine, histamine, and immune systems are viewed as interdependently functioning circuits that through dynamic bi-directional dialog are continually informed and adjust output. This point was eloquently stated in 1949 by Walter Hess in the opening few sentences of his Nobel Prize speech. Mediational models shift the research agenda from correlation and the tendency to generate faulty causality inferences when correlations are high (e.g., epidemiology) and to miss important mediating variables when correlations are low.

Acknowledging that autonomic state functions as an intervening variable is the first principle of PVT. This principle transforms research questions and hypotheses, which had previously focused on exploring correlational data to an algorithm that would have predictive utility in explaining the dynamic adaptive adjustment of autonomic state. An algorithm, through extensive scientific investigation, could lead to a better understanding of conditions and individual differences that would document the impact of enhanced or dysfunctional autonomic support on homeostatic processes including health, growth, restoration, and sociality.

### 17.3 Principle 2. three neural circuits form a phylogenetically ordered response hierarchy that regulate autonomic state adaptation to safe, dangerous, and life-threatening environments

Principle 2 has been thoroughly described in the sections above. PVT emphasizes that three neural circuits regulating autonomic state are important determinants of the biobehavioral algorithm that enable autonomic state variations to predictably support different adaptive functions. A careful literature review documents that brainstem nuclei regulating the ANS follow a phylogenetically ordered sequence which is initiated with in ancient vertebrates and through the process of evolution was modified and repurposed in mammals. PVT is mammalian-centric and focused on identifying and describing the biobehavioral scripts produced by a hypothetical brainstem algorithm that would optimize survival in humans. The phylogenetic sequence is initiated by a dorsal vagus, followed by a spinal sympathetic system, and finally with the ventral vagus. By identifying the biobehavioral scripts of each of these circuits, we become appreciative of the efficiency of the three neural circuits in an attempt to optimize survival in response to signals of safety, danger, and life threat.

The scripts are helpful in identifying when the ANS is in a state that supports homeostatic functions (health, growth, restoration, and sociality), when it supports the metabolically costly states requiring fight and flight behaviors, and when it supports threat reactions of immobilization (death feigning) that may not support the organism's oxygen needs. Identification of the three circuits provides a neurophysiological basis to explain the mechanisms through which each ANS state supports different behaviors and experiences. As emphasized in Principle 4, the biobehavioral consequences of this ventral migration of cardioinhibitory neurons in the brainstem provides an organizing principle to understand that the neural regulation of the ANS in humans is an enabler of sociality [33,34].

### 17.4 Principle 3. in response to a challenge, the ANS shifts to states regulated by circuits that evolved earlier consistent with the Jacksonian principle of dissolution [30], a guiding principle in neurology

There are hundreds, if not thousands, of peer reviewed publications documenting the involvement of ventral vagal regulation of the heart (monitored through metrics of HRV and especially RSA). In these studies, systematic withdrawal the ‘newer’ ventral vagal calming pro-homeostatic actions occur during contexts requiring the recruitment of metabolic resources to move including fight-flight behaviors, mental and physical illness, psychological challenges (e.g., mental effort, sustained attention) and the anticipation to move when feelings of threat are experienced. In contrast, feelings of safety seem to parallel an ANS in a more flexible state that enables movement to be integrated with other forms of co-regulation involving attributes of the social engagement system. Thus, providing the autonomic substrate that would discriminate play from defense.

Although a much smaller literature, there appears to be documentation of an immobilization defense response to signals of life threat that trigger a death feigning response (e.g., mouse in the jaws of a cat, rats [10]) that include a reduction in neuromuscular tone and the associated reduction in autonomic activation that has been hypothetically linked to dorsal vagal influences on heart rate, contractility, and gut motility. It should not be a surprise that individuals whose nervous systems have responded as if they were under life threat, frequently have a retuned ANS with features of autonomic dysregulation especially gut problems. Potentially, gut symptoms may be a product of a dampened ventral vagal circuit that resulted in a vulnerability to the dorsal vagal circuit being recruited in defense [68,81,82].

Principle 3 is helpful in redefining psychological constructs of stress and anxiety as physiological states that support defense. Succinctly PVT would define stress, anxiety, or any threat related experience as a disruption in homeostatic function. Although PVT was initially focused on transitory acute challenge related changes in autonomic state, the theory provides insights into chronic states and illnesses. It proposes that the resilience of the ANS may be dampened or retuned to be chronically locked into states of defense. Hypothetically this may be the consequence of a life-threatening experience with symptoms that would persist even when the body was not physically injured or even after the body healed. This sequence appears to reflect a nervous system, which is adaptively reluctant to relinquish its defenses. Examples of this have been reported as a consequence of severe adversity history in which the regulation of the ANS has transitioned from an algorithm supporting homeostatic functions to one that supports defense at all costs. Trauma therapists are familiar with these observations in which patients react defensively when socially engaged through proximity and even eye contact. In these cases, the nervous system is optimizing defense at the expense of supporting the homeostatic processes of health, growth, restoration, and sociality.

### 17.5 Principle 4. ventral migration of cardioinhibitory neurons leads to an integrated brainstem circuit (ventral vagal complex) that enable the coordination of suck-swallow-breath-vocalize, a circuit that forms the neurophysiological substrate for an integrated social engagement system

PVT is interested in the process through which ventral migration of cardioinhibitory neurons became integrated in the regulation of the striated muscles of the face and head. This is a critical event enabling mammals to nurse and to signal the caregiver. Interestingly, this system seems to have provided the core mechanisms that enabled mammals to co-regulate and to communicate with conspecifics. PVT speculates that the ventral migration paved the path for mammalian sociality, which enabled co-regulation and trust to be a highlight of human behavior as well as the system that is most challenged when the ANS shifts into a state of defense. It is a system that can literally be monitored in real time by studying heart rate patterns in preterm human newborns (see above). The social engagement system, through the expression of autonomic state of calmness in vocalizations and facial expressions is a potent stimulus through neuroception (see Principle 5) for mammals to down regulate threat reactions and become a portal to signal safety to conspecifics. This intuition is frequently understood by therapists, parents, teachers, friends, and pet owners as they use their voice and gestures projecting their own calm state to calm others.

### 17.6 Principle 5. neuroception: reflexive detection of risk triggers adaptive autonomic state to optimize survival

The construct of an ‘algorithm’ was selected to emphasize that the autonomic signatures related to navigating in contexts that are safe, dangerous, or life threatening are basically reflexive brainstem scripts. Neuroception is the hypothetical process through which these scripts are triggered. According to PVT these scripts reside in the brainstem area that regulates foundational survival mechanisms. In humans and other social mammals, these scripts are triggered by higher brain structures that process information outside of awareness. By being reflexive these processes are unimpeded by intentionality and cognitive appraisal. Adaptively, if they were, decisions would be slow and potentially tentative, and survival might be compromised. To emphasize the independence of these processes distinct from awareness and intention, PVT introduced the construct of neuroception, which ‘detects’ and triggers foundational survival mechanisms. Since neuroception does not involve perception or appraisal of causality, neuroception cannot be modified through cognitive channels. Neuroception, including assumed relationships with temporal cortex and periaqueductal gray, has been described elsewhere [78,79] and summarized above.

## 18 Current status of PVT

The foundation of PVT is based on the listed principles (see Table 1) extracted from an accepted scientific literature. The validity of the theory should be based on the utility of these principles to provide plausible explanations in clarifying human experience. The theory is informed by several disciplines (e.g., evolution, comparative neuroanatomy, cardiopulmonary neurophysiology), although PVT was not structured to answer questions or test hypotheses relevant to these disciplines. The theory should be evaluated based on the scientific questions that stimulated the quest to understand the ‘vagal paradox’ as framed in the presentation of the theory and the scientific foundation from which the above five principles have been extracted. The theory focuses on the role of the ANS as an intervening variable and explores the impact of disruptive challenges on homeostatic functions. The model embedded in the theory, by emphasizing the ANS as an intervening variable, expands clinically relevant research questions from testing cause and effect hypotheses to questions of how the neural regulation of ANS mediates reactivity and recovery to challenges. Thus, this mediational model could be conceptualized as a functional neural algorithm.

**Table 1:** Polyvagal Theory Principles.

**Principle 1.** Autonomic state functions as an intervening variable.
**Principle 2.** Three neural circuits form a phylogenetically ordered response hierarchy that regulate autonomic state adaptation to safe, dangerous, and life-threatening environments.
**Principle 3.** In response to a challenge, the ANS shifts to states regulated by circuits that evolved earlier consistent with the Jacksonian principle of dissolution [30], a guiding principle in neurology.
**Principle 4.** Ventral migration of cardioinhibitory neurons leads to an integrated brainstem circuit (ventral vagal complex) that enables the coordination of suck-swallow-breath-vocalize, a circuit that forms the neurophysiological substrate for an integrated social engagement system.
**Principle 5.** Neuroception: Reflexive detection of risk triggers adaptive autonomic state to optimize survival.

Given the strong scientific foundation, there have been few criticisms in the scientific literature. As stated above, almost 30 years ago the initial presentation of the theory was structured as a challenge to colleagues to expand, refine, or refute features of the presentation with an optimistic and collaborative goal of gaining a better understanding of how autonomic state was related to human experience. In general, the scientific and clinical community welcomed PVT as an innovative perspective linking the ANS with human health and experience. Consistent with this acceptance, during this period several peer-reviewed grants from the National Institute of Health supported my research exploring the clinical relevance of PVT. However, there were a few, who criticized the theory without accurately representing the theory.

Their criticisms evolved into a classic *strawman* argument that the theory did not have a scientific basis. The criticisms were not based on disagreements of interpretation but were formulated as statements of falsification of the theory. Nor were their criticisms relevant to the basic questions and hypotheses related to the theory. Investigation of their criticisms has identified two important points: 1) their criticisms were based on inaccurate representations of the theory and 2) their criticisms were irrelevant to the theory and the questions that stimulated the structuring of the theory. Then consistent with the classic structure of a *strawman* argument their misrepresentations were repeatedly presented as evidence that the theory was untenable. These arguments were initially seeded about 20 years ago [59]. A response followed [83], attempting to correct their misunderstandings of the theory including documenting that their proposed ‘replacement’ theory for PVT was a paraphrased extraction of features of PVT without acknowledgement. Their *strawman* argument dissolves once the conjectures about PVT are documented as false. A few examples of their many misrepresentations of PVT are summarized below. Unfortunately, these same scientists continue to misrepresent the theory and to argue points unrelated to the principles embedded in PVT ([36,84]).

Now approximately 30 years after the initial presentation of the theory, the current paper attempts to clarify PVT by providing accessible principles and an updated review of the supporting scientific literature. An additional goal of the paper is to explicitly document that the primary critiques of PVT are focused on a neurophysiology that is not consistent or even relevant to the theory. It is hoped that future scientific dialog and debate instead of being *strawman* arguments will more accurately represent PVT and challenge it through more traditional strategies such as hypothesis testing and alternative explanations of the literature.

**PVT defines RSA as a mammalian form of cardio-respiratory coupling.** In 2005 Taylor's research group published an early paper misrepresenting the theory [85], “Does Respiratory sinus arrhythmia occur in fishes?” Below is a quote from this paper.

*In addition to these data on fish, it has been observed that many amphibians and reptiles, characterized as breathing discontinuously, show close correlations between the onset of a bout of breathing and an instantaneous tachycardia, implying overriding central nervous integration of their cardio-respiratory systems (Burggren 1987). However, Porges (1995) proposed that cardio-respiratory coupling is restricted to mammals. p.484*

**PVT does not assume that cardio-respiratory coupling is restricted to mammals**. The Taylor group has repeated their inaccurate assumption that mammalian RSA is equivalent to other forms of non-mammalian respiratory-heart rate interaction. By not acknowledging the unique neuroanatomical and neurophysiological differences between RSA and respiratory-heart rate in other vertebrates, they assume that the documentation of any form of respiratory-heart rate interaction in species other than mammals would document that PVT is inaccurate. Below is a quote from their paper on rattlesnakes in which PVT is misrepresented [86].

*This has led to the conclusion that RSA does not exist in non-mammalian vertebrates and forms the basis of the polyvagal theory (Porges, 2003). p. 2635*

*This [the observation of a respiratory pattern in the heart rate pattern in a rattlesnake] data refutes the proposition that centrally controlled cardiorespiratory coupling is restricted to mammals, as propounded by the polyvagal theory of Porges (Porges, 1995; Porges, 2003). p. 2635*

These statements are blatantly false. Since they infer that if cardio-respiratory coupling cannot be restricted to mammals, this becomes a critical flaw in PVT. Following their logic, observations of heart rate-respiratory coupling in other vertebrate species would be inconsistent with the theory. Their convoluted logic works well ONLY if the term RSA is redefined to be inclusive of all forms of heart rate-respiratory coupling observed in vertebrates. Then since PVT uses the construct of RSA, they could assume that any statement regarding the uniqueness of RSA as being mammalian would be false. This inaccurate argument continues to be expressed (see Ref. [36]. Unfortunately, they missed two important points about the relationship between RSA and PVT: **1) the specific vagal pathways mediating cardio-respiratory coupling in mammals (i.e., RSA), unlike their ancestral vertebrates, originate in the ventral vagus, and 2) RSA is a portal to the function of the ventral vagus, enabling the testing of polyvagal-informed hypotheses, but is not a foundational construct of the theory.**

I responded [83] to one of their early misrepresentations [59] in the quote below.

*This statement is perplexing, since the specific restriction of cardiorespiratory coupling to mammals was not stated in the Polyvagal Theory. Moreover, as discussed in the commentary, from the Polyvagal perspective, RSA is a uniquely mammalian cardiorespiratory interaction because it is dependent on the outflow of the myelinated vagus originating in the nucleus ambiguus. This does not preclude cardiorespiratory interactions involving the unmyelinated vagus originating in the dorsal motor nucleus of the vagus in other vertebrates.*

**Myelinated cardioinhibitory vagal fibers originating in the nucleus ambiguus is a defining feature of the phylogenetic transition from ancient, long extinct reptiles to mammals**. Taylor's research group, although acknowledging that in mammals myelinated cardioinhibitory vagal fibers predominantly originate from the nucleus ambiguus, continued to express a misunderstanding of the role of myelinated vagal fibers in PVT. They have argued that the identification of myelinated cardioinhibitory vagal pathways in species other than mammals disproves the theory. In an inaccurate representation of PVT, they published a paper entitled “Cardiorespiratory interactions previously identified as mammalian are present in the primitive lungfish” [49]. Below is a quote from that paper which inaccurately represents PVT.

*He [Porges] identifies a phylogenetic progression from the regulation of the heart by endocrine communication, to unmyelinated nerves, and finally to myelinated nerves found exclusively in mammals and persists in stating that “only mammals have a myelinated vagus,” linking this to the evolution of the NA [nucleus ambiguus]. The present study reveals that the mechanisms he identifies as solely mammalian are undeniably present in the lungfish that sits at the evolutionary base of the air-breathing vertebrates*. *p. 7*

Note that PVT focuses on the role of myelinated vagal pathways that originate in the ventral vagus (i.e., nucleus ambiguus), which in mammals have a respiratory rhythm in contrast to the pathways originating in the dorsal motor nucleus of the vagus. Recently, a similar argument was used by citing a study documenting a myelinated vagal pathway originating in the dorsal motor nucleus of the vagus in sheep [84], although the study did not identify the function of these fibers or document that the functional output was coupled with respiration.

In the above quotes we witness how the concept of RSA has been generalized as a term for heart rate-respiratory coupling across vertebrate species. We also see how previous statements about the unique mammalian features of RSA can be reconstrued and distorted. In this manifestation, the word myelinated is repurposed from being associated ONLY in mammals with cardioinhibitory pathways exhibiting a respiratory pattern originating in the ventral vagal nucleus to a general feature of cardiorespiratory interaction independent of nucleus of origin (i.e., either ventral or dorsal motor nucleus of the vagus) and finally independent of function.

Taylor's group has continued using their redefinition of RSA in inaccurate representations of the theory.

*Several authors have shown that HRV related to respiration is present in species of amphibians, reptiles [for example, rattlesnakes], and birds [ducks and shearwaters]. Thus, the repeated contention, central to the polyvagal theory, that the structural and functional bases of RSA are solely mammalian is clearly fallacious. (*[49]*, p 8).*

*These findings do not provide support for Porges' so-called ‘polyvagal theory’, in which the author claims respiratory sinus arrhythmia and its basis in parasympathetic control of the heart is solely mammalian* [86]

*Nevertheless, the promoter of the polyvagal theory recently stated that: ‘only mammals have a myelinated vagus’* [86]

Taylor's group, while inaccurately representing PVT, failed to recognize the descriptive features of the theory in which RSA in mammals is dependent on myelinated cardioinhibitory vagal pathways originating in the ventral vagus and NOT unmyelinated (or potentially myelinated) cardioinhibitory vagal pathways originating in the dorsal vagus. This descriptive statement does not preclude the identification of myelinated cardioinhibitory vagal fibers in vertebrate species. The statement emphasizes the distribution in mammals of myelinated cardioinhibitory vagal fibers that predominantly originate in the ventral vagal nucleus and NOT the dorsal motor nucleus of the vagus. The ventral migration of cardioinhibitory vagal neurons culminating in the clustering of these neurons in the ventral vagus that is mapped out in phylogeny has been documented since the late 1970s [38].

Their proposition that PVT states that only mammals have myelinated vagal pathways is inaccurate. Even in the original PVT paper [1] there is a strong emphasis that the primary source of myelinated cardioinhibitory vagal pathways in mammals originate in the ventral vagal nucleus.

*The Polyvagal Theory argues that [in mammals] the vagal fibers from the DMNX and NA are distinguishable in structure and function. Specifically, it has been argued that the vagal efferent fibers from the NA [nucleus ambiguus is the ventral vagal nucleus] are myelinated and contain a respiratory rhythm and the vagal efferent fibers from the DMNX [dorsal motor nucleus of the vagus] are unmyelinated and do not express a respiratory rhythm. (Porges, 1995, pp 307–308* [1]*).*

This statement is consistent with current neurophysiological research [5] describing vagal pathways in mammals and does not contradict reports of a myelinated vagal pathway from the dorsal vagus in lungfish or the citation of one occurring in sheep. Although interesting, these findings are irrelevant to the theory and not a test of it.

Taylor and colleagues have also questioned the assumption that the dorsal motor nucleus of the vagus is an evolutionarily older structure than the ventral vagus. It has been reliably documented that prior to mammals the prominent cardioinhibitory vagal neurons in vertebrates originated in the dorsal motor nucleus of the vagus. Thus, it is indisputable that estimating an evolutionary timeline through phylogeny, cardioinhibitory neurons originated first in the dorsal motor nucleus of the vagus and then consistent with Taylor's own work [36] migrated ventrally. In the earliest (now extinct) mammals this ventral migration was sufficiently complete to embed cardioinhibitory functions with activities of branchiomotor neurons (i.e., special visceral efferent pathways) that regulate the striated muscles of the face and head promoting ingestion (e.g., nursing) and social communication via facial expression and vocalizations.

Inexplicably, it has been argued [84] that a repurposing of the ANS that would support sociality is an inaccurate assumption - a conclusion that would be inconsistent with the critical role of nursing in mammals as a social behavior, and its dependence on the ventral migration of cardioinhibitory neurons. Or, more generally, how feeding is used to tame and calm (i.e., socialize) domesticated mammals of several species. Grossman, a collaborator of Taylor, supports this point by citing a paper in a special issue of Biological Psychology [84], which he edited. The paper argues that PVT is unappreciative of the social behavior of nonmammalian vertebrates [87]. The authors argue that PVT inappropriately describes reptiles as being asocial, since reptiles have social behaviors. These criticisms are irrelevant to PVT, which is mammal centric. Sociality through a Polyvagal lens focuses on the transformative qualities of social behavior expressed in mammals such as mother-infant interactions and other co-regulatory behaviors that have profound impact on calming autonomic state to optimize homeostatic functions. This continuation of the *strawman* argument is not only applying theory to questions in another discipline, but to a question (i.e., social behavior in reptiles) that has been explicitly stated to be outside the scope of the theory.

In the quote below Taylor and his group [88] acknowledge the theory's definition of mammalian RSA as being restricted to the ventral vagus and myelinated cardioinhibitory fibers. However, in a convoluted way they blur the anatomical and functional distinctions of the dorsal and ventral vagal nuclei that have occurred through evolution, by postulating a primitive ventral vagus without acknowledging functional limitations of this hypothetical anatomical structure [89].

*The “polyvagal theory” has suggested that the beat-to-beat control of fH [heart rate frequency] that generates RSA is restricted to mammals, which have evolved myelinated vagal pathways that originate in the NA [nucleus ambiguus] (Porges 2003; Porges* et al. *2003). However, CRS [cardiorespiratory synchrony] has been reported in both resting dogfish (Taylor 1992) and hypoxic trout (Randall and Smith 1967), and both species have CVPN [cardiac-specific preganglionic neurons] located both in the DVN [dorsal motor nucleus of the vagus] and in a ventrolateral location outside the DVN that may constitute a primitive NA [nucleus ambiguus] (Taylor 1992).*

The generalization of common mechanisms underlying heart rate-respiration interactions across vertebrate species has its limitations. Evolution repurposed and modified how the mammalian autonomic nervous system is both structured and functions. If we do not acknowledge the evolutionary repurposing of structures, we would be vulnerable to be criticized as accepting ‘recapitulation’ theory; a disproven theory that assumes that evolution not only preserves structure, but also function.

RSA has historically been used to describe a mammalian heart rate rhythm. It has a history of use that has been agnostic of the heart rate-respiratory interactions of other vertebrates. In fact, Taylor in his earlier papers (i.e., prior to 2000) uses the term RSA only when discussing mammals. Although respiratory-heart rate interactions are highly conserved during evolution and even evidenced in mammals, the underlying mechanisms have been modified through evolution (e.g., Ref. [51]). These points are emphasized in PVT and elaborated in this paper. The foundation of PVT focuses on the structural and functional consequences of mammalian modifications of this highly conserved system. This point was unambiguously stated in the title of the paper introducing PVT [1] - *Orienting in a defensive world: Mammalian modifications of our evolutionary heritage. A polyvagal theory.*

The *strawman* arguments presented above are documented misrepresentations of PVT and not a scientific debate related to the hypotheses and inferences generated by the theory. In deconstructing their *strawman* arguments, we note that their myopic perspective assumes that the theory was developed to answer questions in their areas of interest that are often unrelated to the focus of PVT. This perspective limits them from asking questions relevant to PVT. This does not preclude the relevance of PVT as an explanatory vehicle to interpret their own work. For example, much of Grossman's own research on RSA can be explained by PVT; a point documented in a paper by Grossman and Taylor [59] in which aspects of PVT were paraphrased and presented as a novel model without attribution [83].

Based on these *strawman* arguments there have been unsubstantiated bold criticisms of PVT suggesting that the theory is speculative and not supported by science. Such statements are inconsistent with an immense and expanding literature supporting attributes of PVT. On the surface, the theory has been cited thousands of times as support for research conducted by independent researchers. However, this is a gross underestimation of the explanatory value of the theory. By investigating the literature on autonomic reactivity through a Polyvagal lens, we can explore whether the results of studies can be explained by the principles embedded in PVT, even if PVT was not cited in the study. This strategy was implemented in a systematic review documenting the impact of contemplative practices on ventral vagal tone, which was evaluated from a Polyvagal perspective [90].

As emphasized in the principles, the succinctly outlined phylogenetically ordered hierarchy involving brainstem structures regulating autonomic state provides a plausible road map of human autonomic reactivity by providing examples of biobehavioral features associated with each of the three major autonomic pathways. The simplicity of embedding in PVT this unchallengeable hierarchy with the Jacksonian principle of dissolution has been transformative in explaining biobehavioral consequences of adversity in the treatment of mental health challenges. Moreover, these principles are permeating the treatment of patients in medicine and students in education.

From a Polyvagal perspective, the numerous studies that document or test hypotheses related to autonomic state as an intervening, a response, or an individual difference variable are implicitly testing attributes of PVT. Reviewing the literature through the lens of PVT provides plausible explanations and neurophysiological pathways mediating outcomes. For example, PVT provides plausible explanations of studies that structure protocols to evaluate the following processes.

1. Autonomic state functions as an intervening (mediational) variable (Principle 1),
2. Changes in heart rate and RSA during challenge (Principles 2 & 3).
3. The efficiency of the vagal brake is related to clinical symptoms (Principles 3 & 4).
4. The impact of vagal nerve stimulation on autonomic state regulation and social behavior (Principles 4).
5. Autonomic state biases reactions (neuroception) along a continuum of risk (Principle 5).

## 19 Conclusion

The scientific method seeks to distinguish valid points from conjectures. Theories flourish only if are useful in describing phenomenon that can inform future investigations. Of course, theories must be modified and informed by empirical research and when necessary, replaced by alternative theories that are more effective in explaining naturally occurring phenomena. If we use this as an acceptable standard, then PVT provides a testable model describing how the mammalian autonomic nervous system reacts to threat and safety. The theory specifically provides an understanding of the core features of the mammalian ANS needed to co-regulate and trust others. It also provides insights into the consequences of autonomic state for mental and physical health. Perhaps, most important the theory gives a voice to the personal experiences of individuals who have experienced chronic threat (i.e., trauma and abuse) or illness and structures an optimistic journey towards more optimal mental and physical health. It is this core, described by PVT, that links our biological imperative to connect with others to neural pathways, via neuroception, that calm our ANS. These systems, in the context of mammalian physiology, are foundational processes through which behavioral experiences can lead to sociality and optimal health, growth, and restoration. In the future, without the distractions of *strawman* arguments, there is an optimistic possibility of a more informed level of scientific discourse that would further explore the important relationships between the ANS and human experience that have been highlighted by PVT.

## Funding

This work was supported by gifts to the Traumatic Stress Research Consortium from the Chaja Foundation and the United States Association for Body Psychotherapy.

## Author statement

Stephen W. Porges is the sole author of the manuscript entitled The Vagal Paradox: A Polyvagal. He is solely responsible for the conceptualization, writing, methodology, and representation of all intellectual information described in the manuscript.

## Declaration of competing interest

The authors declare the following financial interests/personal relationships which may be considered as potential competing interests.I receive a royalty from Integrated Listening System/Unyte for licensing the technology in the Safe and Sound Protocol.
