# Corrigendum to’ The vagal paradox: A polyvagal solution’ [Compr. Psychoneuroendocrinology 16C (2023) 100200]

**DOI:** 10.1016/j.cpnec.2024.100233

**Published:** 2024-04-09

**Authors:** Stephen W. Porges

**Affiliations:** a Traumatic Stress Research Consortium, Kinsey Institute, Indiana University, Bloomington, IN, USA; b University of North Carolina at Chapel Hill, Chapel Hill, USA

The two references to ‘paraphrasing’ in this article should, in accordance with the citations given at each point, be read by reference to Dr. Porges’ article in Biological Psychology (https://www.sciencedirect.com/science/article/pii/S0301051106001906) in which Dr. Porges clarifies the meaning of this term and which, for the avoidance of any doubt, is not an allegation of plagiarism or any similar act.

